# Analysis of Chest CT Results of Coronavirus Disease 2019 (COVID-19) Patients at First Follow-Up

**DOI:** 10.1155/2020/5328267

**Published:** 2020-11-01

**Authors:** Lingshan Zhong, Shuo Zhang, Jigang Wang, Xinqian Zhao, Kai Wang, Wenlong Ding, Zhiheng Xing, Jun Shen

**Affiliations:** ^1^Department of Radiology, Haihe Hospital, Tianjin University, Tianjin Institute of Respiratory Diseases, Tianjin 300350, China; ^2^Department of Respiratory, Haihe Hospital, Tianjin University, Tianjin Institute of Respiratory Diseases, Tianjin 300350, China

## Abstract

**Objective:**

To investigate the dissipation and outcomes of pulmonary lesions at the first follow-up of patients who recovered from moderate and severe cases of COVID-19.

**Methods:**

From January 21 to March 3, 2020, a total of 136 patients with COVID-19 were admitted to our hospital. According to inclusion and exclusion criteria, 52 patients who recovered from COVID-19 were included in this study, including 33 moderate cases and 19 severe cases. Three senior radiologists independently and retrospectively analyzed the chest CT imaging data of 52 patients at the last time of admission and the first follow-up after discharge, including primary manifestations, concomitant manifestations, and degree of residual lesion dissipation.

**Results:**

At the first follow-up after discharge, 16 patients with COVID-19 recovered to normal chest CT appearance, while 36 patients still had residual pulmonary lesions, mainly including 33 cases of ground-glass opacity, 5 cases of consolidation, and 19 cases of fibrous strip shadow. The proportion of residual pulmonary lesions in severe cases (17/19) was statistically higher than in moderate cases (19/33) (*χ*^2^ = 5.759, *P* < 0.05). At the first follow-up, residual pulmonary lesions were dissipated to varying degrees in 47 cases, and lesions remained unchanged in 5 cases. There were no cases of increased numbers of lesions, enlargement of lesions, or appearance of new lesions. The dissipation of residual pulmonary lesions in moderate patients was statistically better than in severe patients (*Z* = −2.538, *P* < 0.05).

**Conclusion:**

Clinically cured patients with COVID-19 had faster dissipation of residual pulmonary lesions after discharge, while moderate patients had better dissipation than severe patients. However, at the first follow-up, most patients still had residual pulmonary lesions, which were primarily ground-glass opacity and fibrous strip shadow. The proportion of residual pulmonary lesions was higher in severe cases of COVID-19, which required further follow-up.

## 1. Introduction

In December 2019, a novel coronavirus pneumonia emerged in Wuhan City, Hubei Province, and spread rapidly to other provinces and cities in China and many other countries [1, 2]. As of April 1, 2020, 823,626 cases were diagnosed and 40,598 deaths were reported worldwide [3]. The International Committee on the Taxonomy of Viruses named this new virus as severe acute respiratory syndrome coronavirus 2 (SARS-CoV-2). Like severe acute respiratory syndrome coronavirus (SARS-CoV) and the Middle East respiratory syndrome coronavirus (MERS-CoV) [4, 5], SARS-CoV-2 also caused a severe respiratory infection, and it was named as Coronavirus Disease 2019 (COVID-19) by the World Health Organization (WHO). It is highly contagious and has a long incubation period, thereby posing a serious threat to human life and health [6–8].

The pathogenesis of COVID-19 involves localized or diffuse acute alveolitis and interstitial inflammation [9]. A large amount of serous fluid, fibrinous exudate, and transparent membrane formation can be found in the alveolar cavity. There is significant proliferation of type II alveolar epithelial cells, reduction of alveolar surface active substances, and dilation and congestion of alveolar septal capillaries. Moreover, interlobular septal edema, mucus, and mucus plugs form in the bronchial lumens. In addition, the pulmonary tissues have focal hemorrhage and necrosis, and there is organization or fibrosis of alveolar exudates. The loss of pulmonary surfactant in critically severe patients causes alveolar collapse and complete failure of the gas exchange function of alveoli, leading to acute respiratory distress syndrome (ARDS). The imbalance between the body's anti-inflammatory and pro-inflammatory responses leads to cytokine storm [10, 11], which in turn leads to systemic inflammatory response syndrome (SIRS), septic shock, and multiple organ dysfunction syndrome (MODS).

Chest CT manifestations of COVID-19 patients essentially reflect pathological changes in the lungs. It has been reported that COVID-19 can be divided into four stages according to the pathological course of the disease, namely, early stage (1–4d), progressive stage (5–8d), peak stage (9–13d), and absorption stage (≥14d) [12]. These stages also conform to the characteristics of chest CT images [13–16]: at the early stage, the lesions are relatively limited and distributed primarily in the subpleural space as single or multiple nodules, or plaque-like ground-glass opacities that might be accompanied by interlobular or intralobular septal thickening. As the disease progresses, there is a widespread exudation and consolidation of the lung parenchyma, and there can also be ground-glass opacity, consolidation, and fibrous strip shadow, simultaneously. The lung structure is distorted, even exhibiting a “white lung” appearance in critically severe cases. During the absorption stage, the lesions gradually shrink, their density gradually decreases, and the main manifestations are ground-glass opacity and/or fibrous strip shadow.

The follow-up period of moderate or severe patients after discharge is still theoretically the absorption stage, and the characteristics of chest CT images are also associated with it. However, there is little chest CT imaging data for the prognosis of COVID-19 patients. In this study, a retrospective analysis of the CT imaging findings of 52 patients with COVID-19 in our hospital at the last time of admission and the first follow-up after discharge was conducted in order to investigate the dissipation and outcomes of residual pulmonary lesions in COVID-19 patients and to improve the awareness of this emerging disease among the medical personnel.

## 2. Materials and Methods

### 2.1. Clinical Data

From January 21 to March 3, 2020, a total of 136 COVID-19 patients were admitted to our hospital. Inclusion criteria included the following: (a) According to the “Diagnosis and Treatment Protocol for Coronavirus Disease 2019 (trial version 7)” issued by the National Health Commission of the People's Republic of China [9], patients diagnosed with COVID-19. (b) Upon admission, according to the diagnosis by the Tianjin COVID-19 medical treatment expert group, the patients were clinically classified as moderate or severe. (c) After discharge, the patients completed the first follow-up CT examination. Exclusion criteria included the following: (a) Mild COVID-19. Patients had positive nucleic acid test, mild clinical symptoms, and no abnormalities in the chest CT. (b) Critically severe COVID-19. There were relatively fewer cases, and the patients had not been discharged or had not been followed up after discharge. (c) The patients had a previous history of lung disease, malignancy, or lung surgery. (d) The patients died during hospitalization. (e) Clinical classification of the patient during hospitalization was worse than that on admission; the moderate cases progressed to severe or critically severe cases, and the severe cases to critically severe cases. (f) Chest CT examination at the last time of admission indicated that pulmonary lesions had completely dissipated. (g) Children with COVID-19. There were only 3 cases in the moderate group and none in the severe group.

**Figure 1:**
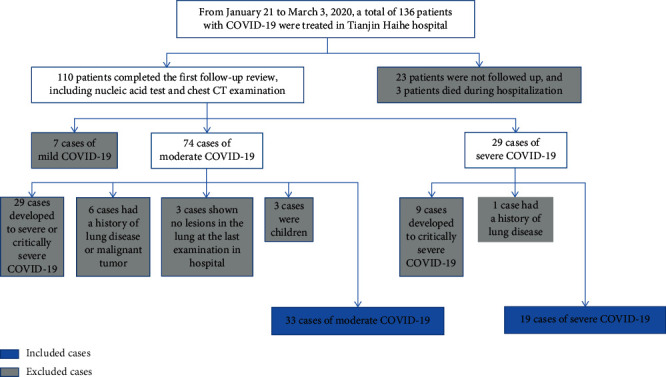
Flow chart of inclusion and exclusion for COVID-19 patients in this study.

Ultimately, 52 patients who recovered from COVID-19 were included in this study, including 33 cases of moderate COVID-19 and 19 cases of severe COVID-19 (Figure 1). Clinical and laboratory data were obtained from a detailed medical record review conducted by 3 senior radiologists using a standardized form. The following clinical data of the patients were assessed: gender, age, symptoms at admission (including fever, dry cough, fatigue, headache, vomiting, abdominal pain, and diarrhea), previous concomitant diseases (including hypertension, diabetes mellitus, heart disease, and cerebrovascular disease), and concomitant diseases developed during hospitalization (such as electrolyte metabolism disorders, hypokalemia, leukocytopenia, anemia, hypoproteinemia, liver dysfunction, renal dysfunction, gastrointestinal dysfunction, stress gastritis, and ketosis). Information regarding the physical examination at admission was also evaluated, including the heart rate, body temperature, and blood pressure. Regarding the laboratory data, the white blood count, lymphocytes, and C-reactive protein (CRP) obtained at admission were assessed.

### 2.2. Examination Method

Scanning was performed using a Canon 64-slice spiral CT scanner (Aquilion Prime 128, Canon Medical Systems, Otawara, Japan). In order to ensure the accuracy of the breathing phase in the collected data, the subjects were trained in deep inspiratory breath hold before scanning. The subjects were placed in a supine position, asked to hold their head with both hands, and then advanced head-first into the scanner. A whole-lung spiral CT scan was performed from the apex to the base of the lungs during deep inhalation while holding breath: tube voltage: 120 kV, automatic tube current modulation, scan speed: 0.5 s/r, matrix size: 512 × 512, collimation width: 64 × 0.5 mm. Image reconstruction was performed using the FC30 and FC52 algorithms with a reconstruction slice thickness of 1.0 mm and a reconstruction interval of 0.8 mm.

### 2.3. CT Evaluation

The chest CT imaging data of COVID-19 patients at the last time of admission and the first follow-up after discharge were retrospectively analyzed, including: primary manifestations (ground-glass opacity, consolidation, fibrous strip shadow), concomitant manifestations (interlobular and/or intralobular septal thickening, subpleural curvilinear line, traction bronchiectasis), and the degree of residual lung lesion dissipation.

Based on the Fleischner Society pulmonary lesion recommendations for the classification of CT signs [17], the imaging feature analysis was to count the frequency of signs in the CT images of each patient. Chest CT images of each patient were independently analyzed by 3 senior radiologists who only knew that the patients had COVID-19. The image analysis data of the 51 patients were consistent among the radiologists. There was only one patient without consensus. For this patient, one radiologist believed that there was no residual COVID-19 lesion in the lung at the first follow-up, and the other two radiologists thought that the fibrous strip shadow was a residual lesion. By reviewing all imaging data of this patient, they considered that the fibrous strip shadow was an old lesion, which was irrelevant to COVID-19.

The three radiologists separately used the Vitrea workstation Lung CT analysis software to measure the volume, and the average of the three measurements was the final result. The percentage of lung involvement at the first CT after symptom onset (*P*_first_) was calculated by measuring the ratio of the total lesion volume (*V*_first_) to the bilateral lung volume (*V*_lung_) (Equation (1)). It was used to assess the initial disease burden of COVID-19 patients and classified as follows: 0∼5%, 5%∼10%, 10%∼20%, 20%∼30%, and 30%∼40%. By measuring the ratio of the total lesion volume at the last time of admission (*V*_last_) to that of the first follow-up after discharge (*V*_follow-up_), the degree of lesion dissipation (*D*_dissipation_) was calculated as Equation (2), and it was classified as complete dissipation, significant dissipation (≥50%), slight dissipation (<50%), or no dissipation.

(1) $$\begin{array}{c} P_{\text{first}}=\frac{V_{\text{first}}}{V_{\text{lung}}}\times 100\%, \end{array}$$

(2) $$\begin{array}{c} D_{\text{dissipation}}=\frac{V_{\text{last}}-V_{\text{follow}-\text{up}}}{V_{\text{last}}}\times 100\%. \end{array}$$

### 2.4. Statistical Analysis

Statistical analysis was performed using SPSS 19.0. Measurement data subject to normal distribution were expressed as $\bar{X}$ ± *S* and were performed using the *t*-test. The measurement data that did not follow normal distribution were expressed as *M* (P_25_, P_75_) and were performed using the Mann–Whitney U test. Count data were expressed in terms of the relative number (ratio and rate) and were performed using the chi-square test or the Mann–Whitney U test. Differences with *P* < 0.05 were considered statistically significant.

## 3. Results

### 3.1. Characteristics of 52 Patients with COVID-19 after Symptom Onset

The clinical data, laboratory data, and pulmonary involvement range of 52 patients after symptom onset is given in Table 1. Body temperature, CRP level, and the range of pulmonary involvement after symptom onset were significantly different between the moderate group and the severe group (*Z* = −2.141; *Z* = 2.062; *Z* = -2.288, *P* < 0.05). Patients with severe COVID-19 had higher body temperature and CRP level, and a wider range of pulmonary involvement after symptom onset. Moderate patients mainly used symptomatic support treatment, including antiviral, phlegm, improving immunity, strengthening nutrition, and oxygen therapy. On the basis of symptomatic treatment, severe patients should actively prevent and cure complications, treat basic diseases, prevent secondary infection, and provide timely organ function support.

### 3.2. Chest CT Manifestations at the Last Time of Admission and the First Follow-Up after Discharge

**Figure 2:**
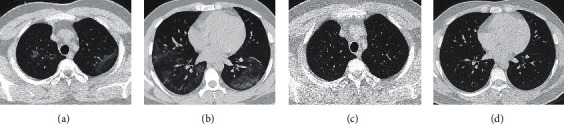
A 29-year-old male patient with a moderate case of COVID-19. (a, b) Ground-glass opacities with unclear boundaries were seen in multiple lobes of both lungs on day 14 after onset of the disease. (c, d) On day 36 after onset and day 20 after discharge, pulmonary lesions dissipated completely.

The time from symptom onset to the last CT of admission was 9 days∼31 days (mean: 18.40 days ± 5.69 days). The time to the first follow-up CT was 29 days∼62 days after symptom onset (mean: 39.60 days ± 5.96 days) and 11 days ∼ 34 days after discharge (mean: 19.71 days ± 4.08 days). Compared with the CT manifestations at the last time of admission (Table 2), the first follow-up results showed that 30.77% (16/52) of COVID-19 patients recovered to a normal chest CT presentation (Table 3, Figure 2). CT findings returned to normal in 42.42% (14/33) of the moderate group and 10.53% (2/19) of the severe group, and the difference between the two groups was statistically significant (*χ*^2^=5.759, *P* < 0.05).

**Figure 3:**
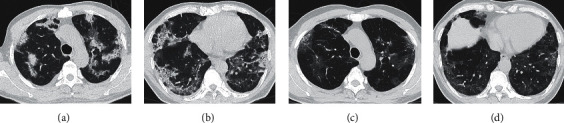
A 55-year-old male patient with a severe case of COVID-19. (a, b) Consolidations and ground-glass opacities were seen in multiple lobes and segments of both lungs on day 25 after onset of the disease. The density was not uniform, and there was an evidence of air bronchograms and intralobular septal thickening. (c, d) On day 47 after onset and day 21 after discharge, ground-glass opacities and fibrous strip shadows were seen in multiple lobes and segments of both lungs. Consolidations dissipated and the intralobular septal thickening was milder compared to the last CT of admission. The total volume of pulmonary lesions was measured and calculated through two CT scans, and the pulmonary lesions were dissipated to a slight degree.

Chest CT imaging of 69.23% (36/52) of COVID-19 patients still revealed residual lesions in the lungs (Table 3, Figure 3). The main manifestation of the residual lesions was ground-glass opacity (33/36, 91.67%), which was localized or distributed in multiple lobes and segments and had low density, lack of homogeneity, and unclear boundaries. Fibrous strip shadow was more common during a pulmonary lesion dissipation (19/36, 52.78%) and presented as strips of different length and thickness, with some adhering to and pulling on the pleura. Consolidation was uncommon in the residual pulmonary lesions (5/36, 13.89%) and presented as nodular and patchy high-density shadows that were markedly smaller or dissipated compared to those in the previous examinations. Three patients exhibited interlobular and/or intralobular septal thickening presenting as thin linear shadows at the periphery of the lungs and reticular patterns within the lungs. These gradually became thinner and fewer as the lesions dissipated. Subpleural curvilinear line, which manifested as thin linear shadows 1 cm below and parallel to the pleura, were found in 5 patients. Traction bronchiectasis, which manifested as a localized columnar extension of the bronchi in the outer band of the lungs, was found in 4 patients. Some patients exhibited more than one of these signs.

### 3.3. Relationship between Residual Pulmonary Lesions and Clinical Classification of COVID-19

The proportion of residual pulmonary lesions in severe cases of COVID-19 was 89.47% (17/19), which was higher than the proportion of 57.58% (19/33) in moderate cases, and the difference between the two groups was statistically significant (*χ*^2^=5.759, *P* < 0.05) (Table 3).

### 3.4. Relationship between Degree of Residual Lesion Dissipation and Clinical Classification of COVID-19

At the first follow-up, residual pulmonary lesions dissipated to varying degrees in 47 cases and remained unchanged in 5 cases. There were no cases of increased numbers of lesions, enlargement of lesions, or appearance of new lesions. The dissipation of residual pulmonary lesions in moderate patients was better than in severe patients, and the difference between the two groups was statistically significant (*Z* = −2.538, *P* < 0.05) (Table 4).

## 4. Discussion

COVID-19 is spreading all over the world, and the situation in some countries and regions is still grim. Although there is an increase in the number of patients who have recovered from COVID-19, due to the hysteresis of imaging manifestations, most patients still have various pulmonary lesions at discharge. Therefore, the dynamic changes and outcomes of COVID-19 residual lesions are of great concern to clinicians and radiologists.

In this study, the main clinical manifestations of COVID-19 patients were fever (98.08%) and dry cough (48.08%). We found that there were differences in some clinical and radiological data after symptom onset between patients with moderate and severe COVID-19, including body temperature, CRP level, and range of pulmonary involvement. The severe COVID-19 patients had statistically higher body temperature and CRP levels and a wider range of pulmonary involvement after symptoms appeared than the moderate ones. The high body temperature indicated that the immune system of severe patients was highly activated. The increased values of CRP in severe patients might be related to cytokine storm induced by virus invasion, which was supported by a recent study [13]. We believed that the wider the pulmonary involvement, the greater the burden of disease, and the more the likelihood of residual lesions after treatment. Chau et al. [18] found that SARS patients with a wider range of lung involvement were more likely to receive mechanical ventilation treatment and had a higher risk of death. In this study, patients with a history of pulmonary disease and malignancy were excluded in case selection, while other previous concomitant diseases and concomitant diseases developed during hospitalization showed no statistically significant differences between the two groups.

A retrospective analysis of the chest CT findings of 52 patients with COVID-19 at the last time of admission and the first follow-up after discharge found that 90.38% (47/52) of the clinically cured patients with COVID-19 had various degrees of pulmonary lesion dissipation. The chest CT manifestations returned to normal in 30.77% (16/52) of COVID-19 patients by about day 20 after discharge. The dissipation process of residual pulmonary lesions were faster and more thorough, suggesting that the pathological basis of COVID-19 was exudative change. It was more common in the moderate group compared to that in the severe group (14/33 vs 2/19). This result indicated that the lesions of the moderate group dissipated faster in the absorption stage, and the time needed for the lesions to reach complete dissipation was shorter. We believed that this might be related to a strong inherent immunity and a relatively milder lung disease in these patients. This study also showed that the range of pulmonary involvement after symptom onset in moderate patients is significantly smaller than that in severe patients, which supported our view.

Residual lesions persisted in the lungs at the first follow-up in 69.23% (36/52) of COVID-19 patients, and the main manifestation was ground-glass opacity, which was significantly reduced in density and extent compared to the ground-glass opacity at the progressive and peak stage of the disease. A gradual absorption and subsidence of the alveolar lumen exudation and alveolar wall capillary congestion at the pathological level with disease progression as well as pathological changes in the residual alveolar walls and other interstitial tissues and interstitial fibrosis might be associated with this observation [19]. In severe cases, the extent of the lesions was larger and more severe, and their dissipation was slower. Consolidation was relatively rare in the residual pulmonary lesions, and most consolidations dissipated significantly at the first follow-up compared to those during hospitalization. Irregular fibrous strip shadow could appear during the dissipation process of lesions, which might first be seen in the progressive stage. Their pathological basis was the proliferation of fibroblasts, vascular endothelial cells, and connective tissue, which ultimately forms a fibrous tissue. Fibrosis is the result of damage repair, and fibrous strip shadow often appears in other viral pneumonias such as those with the SARS-CoV, MERS-CoV [4], and so on. Some researchers [20] believed that changes in the ground-glass opacity, interlobular and/or intralobular septal thickening, subpleural curvilinear line, and traction bronchiectasis were early symptoms of the pulmonary interstitial fibrosis. However, at the first follow-up, the majority of patients presented with ground-glass opacity, and only a small number of patients with interlobular septal and/or intralobular septal thickening, subpleural curvilinear line, and traction bronchiectasis were observed. Fluid, fibrous, or cellular infiltration could cause pulmonary interstitial thickening. These signs suggested that the lesion involved the interstitial of alveolar walls, but it was not equal to fibrosis, which could also be caused by inflammation or edema. These signs could be seen in the early stage of COVID-19 and completely disappear with the improvement. Therefore, these signs were reversible and could not be used as a reliable indicator for the judgment of a pulmonary fibrosis, while long-term changes remained unknown.

We believe that patients in the two groups have different disease conditions and different peak lesion degrees with similar lesion types, so the CT signs in follow-up are similar with difference in degree. As CT follow-up is in the absorption period, referring to the symptoms of viral pneumonia such as SARS [4], the dissipation of residual lesions takes time, and the specific dissipation degree varies with the nature of the lesions. Previous studies used a semiquantitative visual score method to individually evaluate the extent of each lobe lesion and calculate the total score for 5 lobes [21, 22]. In this study, the Canon Vitrea workstation was used to measure the total volume of pulmonary lesions and calculate the dissipation volume ratio of the lesions over two CT examinations. This approach can be used to evaluate the dissipation of lesions more accurately, objectively, and quantitatively. And the results showed that the lesions of moderate patients were absorbed better than those of severe cases, and the difference was statistically significant. This indicated that the lesions of moderate patients were absorbed faster in the absorption stage, suggesting that the residual lesions in follow-up CT of moderate patients were less than those of severe patients, and the ground-glass opacity and consolidation were absorbed well.

There are several potential limitations of this study. Firstly, the sample size was relatively small. Secondly, critically severe patients had a long course of disease, and most of them had not revisited during the study period, so they were not included in the study. Furthermore, studies of serious clinical events such as ventilator-assisted therapy or fatalities had not been conducted. Finally, the follow-up time of this study was relatively short, the long-term recovery effect of residual lung lesions in patients was unknown, and the therapeutic effect and the final outcome remained to be followed up.

## 5. Conclusions

Clinically cured COVID-19 patients had faster dissipation of pulmonary lesions after discharge and more significant short-term recovery effects. The dissipation of pulmonary lesions in moderate patients is better, and the residual lesions in some severe cases can also be fully dissipated. At the first follow-up, most of the patients who had recovered from COVID-19 still had residual pulmonary lesions, which were primarily ground-glass opacity and fibrous strip shadow. The proportion of the residual pulmonary lesions in severe patients was higher than that in moderate patients, which required further follow-up.

## Figures and Tables

**Table 1:** Characteristics of 52 patients with COVID-19 after symptom onset.

Characteristics	Moderate COVID-19 (*n* = 33)	Severe COVID-19 (*n* = 19)	*P*
Clinical date			
** **Gender M/F	16/17	13/6	0.163
** **Age (years)	43.30 ± 13.62	49.21 ± 13.49	0.137
** **Fever	32	19	1.000
** **Dry cough	18	7	0.219
** **Fatigue	9	6	0.741
** **Headache	3	1	1.000
** **Vomiting	1	0	1.000
** **Abdominal pain	2	0	0.527
** **Diarrhea	0	1	0.356
** **Hypertension	7	5	0.937
** **Diabetes mellitus	3	2	1.000
** **Heart disease	0	3	0.083
** **Cerebrovascular disease	0	1	0.356
** **Electrolyte metabolism disorders	8	4	1.000
** **Hypokalemia	4	2	1.000
** **Leukocytopenia	4	2	1.000
** **Thrombocytopenia	0	1	0.356
** **Anemia	1	1	1.000
** **Hypoproteinemia	3	4	0.427
** **Liver dysfunction	16	6	0.235
** **Renal dysfunction	1	1	1.000
** **Gastrointestinal dysfunction	7	1	0.256
** **Stress gastritis	6	4	1.000
** **Ketosis	1	3	0.262
** **Heart rate (bpm)	85 (79, 95)	91 (78, 98)	0.549
** **Body temperature (°C)	36.9 (36.5, 37.5)	37.7 (37.2, 37.8)	0.032
** **Systolic blood pressure (mmHg)	132 (122, 140.5)	136 (123, 145)	0.430
** **Diastolic blood pressure (mmHg)	81 (72, 90)	82 (75, 91)	0.909
Laboratory data			
** **White blood count (10^9^/L)	4.62 (3.66, 5.215)	4.38 (3.20, 5.59)	0.864
** **Lymphocytes (10^9^/L)	1.02 ± 0.35	0.82 ± 0.35	0.052
** **C-reactive protein (mg/L)	9.98 (4.05, 28.82)	36.8 (15.4, 44.8)	0.039
Percentage of pulmonary involvement at the first CT after symptom onset			
** **0∼5%	26	9	0.022
** **5%∼10%	1	1	
** **10%∼20%	3	5	
** **20%∼30%	3	3	
** **30%∼40%	0	1	

**Table 2:** CT manifestations of 52 patients with COVID-19 at the last time of admission.

CT manifestations	Total (*n* = 52)	Moderate COVID-19 (*n* = 33)	Severe COVID-19 (*n* = 19)
Primary manifestations			
** **Ground-glass opacity	51	33	18
** **Consolidation	29	15	14
** **Fibrous strip shadow	36	19	17
Concomitant manifestations			
** **Interlobular and/or intralobular septal thickening	36	17	19
** **Subpleural curvilinear line	19	8	11
** **Traction bronchiectasis	28	13	15

**Table 3:** CT manifestations of 52 patients with COVID-19 at the first follow-up after discharge.

CT manifestations	Total (*n* = 52)	Moderate COVID-19 (*n* = 33)	Severe COVID-19 (*n* = 19)
Normal CT presentation	16	14	2
Abnormal CT presentation	36	19	17
** **Primary manifestations			
** **Ground-glass opacity	33	18	15
** **Consolidation	5	3	2
** **Fibrous strip shadow	19	9	10
** **Concomitant manifestations			
** **Interlobular and/or intralobular septal thickening	3	1	2
** **Subpleural curvilinear line	5	1	4
** **Traction bronchiectasis	4	2	2

**Table 4:** Degree of residual lesion dissipation in 52 patients with COVID-19 at the first follow-up.

Clinical classification	No dissipation	Slight dissipation	Significant dissipation	Complete dissipation
Moderate COVID-19 (*n* = 33)	1	6	12	14
Severe COVID-19 (*n* = 19)	4	4	9	2

## Data Availability

The data used to support the findings of this study are available from the corresponding author upon request.
